# A missense mutation of plastid RPS4 is associated with chlorophyll deficiency in Chinese cabbage (*Brassica campestris ssp. pekinensis*)

**DOI:** 10.1186/s12870-018-1353-y

**Published:** 2018-06-25

**Authors:** Xiaoyan Tang, Yiheng Wang, Yun Zhang, Shengnan Huang, Zhiyong Liu, Danli Fei, Hui Feng

**Affiliations:** College of Horticulture, Liaoning Key Lab of Genetics and Breeding for Cruciferous Vegetable Crops, Shenyang Agricultural University, Shenyang, Liaoning 110866 People’s Republic of China

**Keywords:** Chinese cabbage, Maternal inheritance, Plastome mutant, Plastid ribosomal protein, rRNA processing

## Abstract

**Background:**

Plastome mutants are ideal resources for elucidating the functions of plastid genes. Numerous studies have been conducted for the function of plastid genes in barley and tobacco; however, related information is limited in Chinese cabbage.

**Results:**

A chlorophyll-deficient mutant of Chinese cabbage that was derived by ethyl methanesulfonate treatment on isolated microspores showed uniformly pale green inner leaves and slow growth compared with that shown by the wild type “Fukuda 50′ (‘FT’). Genetic analysis revealed that *cdm* was cytoplasmically inherited. Physiological and ultrastructural analyses of *cdm* showed impaired photosynthesis and abnormal chloroplast development. Utilizing next generation sequencing, the complete plastomes of *cdm* and ‘FT’ were respectively re-mapped to the reference genome of Chinese cabbage, and an A-to-C base substitution with a mutation ratio higher than 99% was detected. The missense mutation of plastid ribosomal protein S4 led to valine substitution for glycine at residue 193. The expression level of *rps4* was analyzed using quantitative real-time PCR and found lower in than in ‘FT’. RNA gel-blot assays showed that the abundance of mature 23S rRNA, 16S rRNA, 5S rRNA, and 4.5S rRNA significantly decreased and that the processing of 23S, 16S rRNA, and 4.5S rRNA was seriously impaired, affecting the ribosomal function in *cdm*.

**Conclusions:**

These findings indicated that *cdm* was a plastome mutant and that chlorophyll deficiency might be due to an A-to-C base substitution of the plastome-encoded *rps4* that impaired the rRNA processing and affected the ribosomal function.

**Electronic supplementary material:**

The online version of this article (10.1186/s12870-018-1353-y) contains supplementary material, which is available to authorized users.

## Background

Mutant lines are ideal resources for discovering novel gene functions and deciphering unknown mechanisms [1, 2]. In higher plants, chlorophyll-deficient mutants are considered important for gaining a better insight into the mechanism of photosynthesis, chlorophyll synthesis, development and differentiation of chloroplast structure, gene functional identification, and nucleo-cytoplasmic interactions [3, 4].

Chlorophyll-deficient mutants can be generally divided into three types: mutants controlled by nuclear genes, mutants controlled by cytoplasmic genes, and mutants controlled by nuclear-cytoplasmic interactions [5]. Most chlorophyll-deficient mutants in different species, such as *Arabidopsis thaliana* [6], rice [7], barley [8], soybean [9], carrot [10], and cabbage [11], are controlled by nuclear genes involved in plastid development and pigment biosynthesis that transferred from the plastids to the nucleus during evolution [12]. Only a small number of chlorophyll-deficient mutants are cytoplasmic mutants [13].

Chlorophyll-deficient mutants have been used as a genetic tool for the initial study of cytoplasmic inheritance [14, 15]. The existence of extranuclear DNA and non-Mendelian inheritance was first reported in leaf color mutants of *Mirabilis jalapa* and *Pelargonium zonale* [14–16]. Ris and Plaut [17] as well as Nass and Nass [18] reported that plastids and mitochondria have their own DNA and independent genetic systems [19]. Cytoplasmic mutations are inherited in a non-Mendelian fashion, which includes three basic modes: maternal inheritance, paternal inheritance, and bi-parental inheritance [20]. Of these, maternal inheritance is the main mode in angiosperms. In *Brassica campestris*, plastids and mitochondria are maternally inherited, whereas in *Brassica napus*, mitochondria are not strictly maternally inherited [21, 22].

A limited number of cytoplasmic inherited chlorophyll-deficient mutants with a non-chromosomal stripe phenotype are mitochondrial mutants generally caused by rearranging [23, 24]. Most cytoplasmic-inherited chlorophyll-deficient mutants are plastome mutants that occur spontaneously [25, 26] or induced by artificial mutagenesis, plastome mutator alleles [27–29], or transformation [30–32]. Previous studies showed that the rate of spontaneous plastome mutations causing chlorophyll deficiency varies between 0.006 and 0.3% in different plant species [33]. Spontaneous plastome mutations include all mutation types, including indels and point mutations. Artificial mutagenesis used to generate plastome mutants is either physical mutagenesis [34] or chemical mutagenesis [35]. N-nitroso-N-methyl-urea (NMU), methyl-nitro-nitrosoguanidine (MNNG), 5-bromo-2′-deoxyuridine (BrdU), and 9-aminoacridine hydrochloride (9AA) have been widely used as chemical mutagens to induce plastome mutations [36–38]. Ethyl methanesulfonate (EMS) mainly induces nuclear gene mutations, but also plastome mutations [39]. Nuclear genes can induce plastome mutations by plastome mutator alleles in frequencies much higher than those of spontaneous mutations [40–42].

Plastome mutants are classified into three categories: those with mutations in genetic system genes, in photosynthesis-related genes, and other genes and conserved reading frames [43]. Numerous plastome mutants with mutations in genetic system genes showed chlorophyll deficiency [13, 42, 44], antibiotic resistance [45, 46], misshapen leaves, or low-temperature tolerance [30].

Genetic system genes, including 62 plastome-encoded genes, 30 tRNAs, 4 rRNAs, and 21 ribosomal protein genes, constitute the largest group in the plastome, which is associated with plastid gene expression [47, 48]. Plastid ribosomal proteins are essential components of the plastid ribosome composed by a 50S subunit and a 30S subunit. The former subunit comprises of 23S rRNA, 5S rRNA, 4.5S rRNA, and 33 plastid ribosomal proteins, whereas the latter subunit comprises of 16S rRNA and 24 chloroplast ribosomal proteins. Additionally, 12 proteins of the 30S subunit and nine proteins of the 50S subunit are encoded by plastid genes [31]. Some plastid ribosomal proteins are essential for plastid translation, plant growth, and plant development, whereas others are non-essential [30, 44, 49]. Plastid ribosomal proteins encoded by plastid genes have been widely studied in tobacco, whereas those encoded by nuclear genes are well studied in *A. thaliana*. The *rps2, rps4, rpl18*, and *rpl20* knockout mutants in tobacco show misshapen leaves, revealing the essential role of plastid proteins in leaf development [30, 44], whereas the *rps15* knockout mutant shows chlorophyll deficiency [31]. In addition, the *prps1, rps5, prsp17*, *prpl11*, and *prpl24* mutants in *A. thaliana* show chlorophyll deficiency and reduced growth, and of these, *prps17* and *prpl24* show rRNA process impairment [50–52]. Therefore, plastid ribosomal proteins play essential roles in plastid translation, plant growth, and plant development. In contrast to simple knock-out analysis of plastid transformation, point mutations or indels in plastome mutants are valuable for elucidating plastid functions [33]. The identification of plastome mutants by the phenotype or using conventional genetic methods is considered unreliable, or it may be due to a mitochondrial mutations secondarily resulting in plastid malfunction. Next-generation sequencing (NGS) provides a relatively reliable and rapid method to directly identify plastome mutants at the DNA level.

In this study, a maternal inherited mutation causing chlorophyll deficiency was identified in Chinese cabbage. The complete plastomes of *cdm* and Fukuda 50 (wild type; ‘FT’) were respectively re-mapped to the cpDNA reference genome of Chinese cabbage [53] using Illumina NGS, and a missense mutation was found in the ribosomal protein S4 (RPS4). The accumulation and processing of plastid rRNAs, including 23S, 16S, 5S, and 4.5S rRNAs, were found to be aberrant in *cdm* compared with those in ‘FT’. Our results suggested that RPS4 is associated with chloroplast rRNA processing and chlorophyll deficiency in *cdm*.

## Methods

### Plant material

In this study, a doubled haploid line rived from the Chinese cabbage variety ‘FT’ was used as a donor for isolated microspore culturing to create a mutant library. In August 2014, ‘FT’ seeds of a doubled haploid line derived from the Chinese cabbage variety Fukuda 50 (‘FT’) were vernalized at 4 °C for 20 d after accelerating germination and then, sown in an aperture disk on a seedbed. Seedlings were re-potted in late September, and the unopened flower buds were used for isolated microspore culturing.

### EMS mutagenesis on isolated microspores

Procedures of microspore isolation, purification, and culture were performed as described previously [54, 55]. Isolated microspore culturing was conducted at the Liaoning Key Laboratory of Genetics and Breeding of Cruciferous Vegetable Crops, Shenyang Agricultural University, China. EMS mutagenesis was performed as described by Huang et al. [56]. In brief, EMS dissolved in B5 medium [57] at a concentration of 0.12% (*v*/v) was filter-sterilized through a 0.22-μm filter membrane. Isolated microspores were washed by B5 medium and centrifuged twice at 120×*g* for 3 min. The precipitate acquired after the first centrifugation was suspended in B5 medium containing EMS (0.12%, v/v) for 10 min. The microspores were re-suspended and cultured in NLN-13 medium [58], incubated at 33 °C for 24 h, and transferred to 25 °C for more than 7 d in the dark. Regenerated plants from cotyledonous embryoids were transferred to Murashige and Skoog (1962) medium [59] and cultured at 25 °C under a 16-h photoperiod for subculture and root induction. The remaining regenerated plants were transplanted in pots after rooting for 20 d.

### Quantification of chlorophyll

Total chlorophyll was extracted as described by Inskeep and Bloom [60]. In brief, fresh inner leaves (0.1 g) from *cdm* and ‘FT’ were respectively selected at the seedling stage (4-week-old) and the flowering stage (8-week-old) and submerged in 80% acetone for 24 h in the dark. The absorbance of the supernatants was recorded at 645 nm and 663 nm using the DU 800 U*V*/Vis Spectrophotometer (Beckman Coulter, Brea, CA, USA).

### Characterization of chloroplast and mitochondrial ultrastructure

The chloroplast and mitochondrial ultra-structures were characterized as described by Lichtenthaler et al. [61] and Huang et al. [56] Fresh inner leaves of *cdm* and ‘FT’ were selected at the seedling stage, cut into small pieces (approximately 1 mm^2^), and fixed in 4% (*v*/v) glutaraldehyde in a 0.1 M phosphate buffer solution (PBS, pH 7.3) at 4 °C for 7 d. The fixed samples were post-fixed in 1% (*w*/*v*) aqueous osmium tetroxide for 4 h and rinsed 3 times in PBS for 5 min each time. The samples were gradually dehydrated with various concentrations of ethanol, and then, impregnated and embedded in Epon812. Ultrathin sections were made using the LKB2088 ultramicrotome (LKB Company, Saffle, Sweden) and double stained with uranyl acetate and lead citrate. Finally, the samples were observed under the H-7650 transmission electron microscope (TEM; Hitachi, Tokyo, Japan).

### Measurement of fluorescence kinetic parameters

Ten 4-week-old *cdm* and ‘FT’ plants with consistent growth were selected for measuring fluorescence kinetic parameters by applying A Fluor Cam Portable ChI/GFP Luminoscope (Handy GFPCam; Eco Tech, Beijing, China) after 20 min of dark adaption using leaf clips. The optional maximal photochemical efficiency of PS II (Fv/Fm), the effective quantum yield of PSII (ФPSII), the photochemical quenching coefficient (QP), and the non-photochemical Chl fluorescence quenching (NQP) were calculated as described by Bilger and Bjorkman [62] and Maxwell and Johnson [63]. Each measurement was performed in triplicate.

### Chloroplast DNA (cpDNA) isolation

At the seedling stage, well-grown *cdm* and ‘FT’ plants were placed at 4 °C for 48 h in the dark to eliminate chloroplast starch granules and reduce the plastid breakage. Fresh leaves collected from three plants were mixed, cleaned with double distilled water, dried on filter paper, and the main nerve was cut off. A 30-g sample was ground to less than 1 mm^2^ in liquid nitrogen. Total cpDNA was isolated using the column Plant chloroplast DNAout kit (TIANDZ, Tianjin, China), according to the manufacturer’s instructions with minor modifications. This experiment was performed on ice, and all vessels and solution were precooled.

### Chloroplast genome re-sequencing by Illumina HiSeq

Purified cpDNA from *cdm* and ‘FT’ was used for constructing an NGS library using the NEBNext Ultra DNA Library Prep Kit for Illumina, according to the manufacturer’s instructions. In brief, 1 μg cpDNA was randomly fragmented to < 500-bp fragments by sonication and treated with End Prep Enzyme Mix, followed by adaptor additions to both ends. Fragments of approximately 410 bp with a 350-bp insert were purified using the AxyPrep Mag PCR Clean-up kit (Axygen, Corning, NY, USA) and then, amplified using P5 and P7 primers. Purified PCR products were validated by the Agilent 2100 Bioanalyzer (Agilent Technologies, Santa Clara, CA, USA) and quantified by the Qubit2.0 Fluorometer (Invitrogen, Carlsbad, CA, USA). DNA libraries were multiplexed, loaded on an Illumina HiSeq instrument, and sequenced by a 2 × 150 paired-end (PE) configuration with the HiSeq Control Software (HCS) + OLB + GAPipeline-1.6 (Illumina) reading sequence information.

Based on the reference cpDNA genome of Chinese cabbage (http://www.ncbi.nlm.nih.gov/genomes/GenomesGroup.cgi?opt=plastid&taxid=3398), cpDNA sequence reads were isolated from the raw sequence reads of mixed nucleus and mitochondrial DNA. The filtered cpDNA sequence reads from *cdm* and ‘FT’ were used for re-sequencing as described by [53].

### Sequence analysis

Based on gene annotation information [64] and nucleotide sequences, PCR primers were designed to verify the mutation site of the candidate gene. Total cpDNA extracted from three *cdm* and plants, respectively, was used as a template. PCR products were purified by a Gel Extraction Kit (Omega Bio-tek, Norcross, GA, USA), introduced into the pGEM®-T Easy Vector (Promega, Madison, WI, USA), and transformed into the JM109 competent cell (Takara, Beijing, China). The recombinant plasmids were sequenced by the Institute of Beijing Genomics, China, and the sequences were aligned using DNAMAN (Lynnon, San Ramon, CA, USA).

### RNA isolation and qRT-PCR

Fresh inner leaves collected from 4-week-old ‘FT’, *cdm*, ‘FT’ × *cdm* and *cdm* × ‘FT’ plants were used for RNA isolation using TRIZOL (Invitrogen). First-strand cDNA was synthesized with random hexamer primers. A 10-fold dilution of cDNA was used for quantitative real time PCR (qRT-PCR) by the Bio-Rad IQ5 Real Time PCR System (Bio-Rad, Hercules, CA, USA) with gene-specific primer sets (Additional file 1: Table S1) and SYBR Green PCR master mix (Takara). The qRT-PCR thermal cycling conditions were as follows: denaturation at 95 °C for 3 min, followed by 40 cycles at 95 °C for 30 s, 60 °C for 30 s, and 70 °C for 30 s. The 2^-△△Ct^ method was used for analyzing the relative gene expression levels as described by Livak et al. [65] and normalized using the house-keeping gene actin and the expression of responsive sample controls. All reactions were quantified in triplicate with three independent biological replicates.

### Northern blot analysis

Total RNA was quantified by Infinite M200PRO (Tecan, Mannedorf, Switzerland). Then, 5-μg aliquots were denatured in RNA loading buffer with ethidium bromide for 15 min at 65 °C, separated in formaldehyde-containing 2% agarose gels, transferred onto Hybond N+ membranes (GE, Boston, MA, USA) by capillary blotting using standard protocols, and fixed by applying a crosslinker (UVP American, Upland, CA, USA). Digoxigenin-labeled plasmid DNA probes were amplified using the PCR DIG Probe Synthesis Kit (Roche, Basel, Switzerland) with gene-specific primers listed in Additional file 1: Table S1 [66], denatured at 100 °C for 5 min, and immerged into a hybridization solution, containing 50% (*v*/v) formamide, 5× saline sodium citrate (SSC), 50 mM phosphate buffer, 0.1% *N-*lauroylsarcosine sodium salt, 7% (m/v) sodium dodecyl sulfate (SDS), and 2% (m/v) blocking reagent (Roche). The membrane was pre-hybridized at 50 °C for 1 h, and then hybridized at 55 °C for 24 h after adding the specific probe in a hybridization oven (UVP American). The membrane was washed in 2× SSC containing 0.1% SDS at 25 °C, and subsequently, washed twice in 0.1× SSC, containing 0.1% SDS, at 68 °C. A 1.5% blocking reagent (Roche) in maleic buffer was applied for immunological detection as described by Huang et al. [67] and the membrane was detected and analyzed by Tano-5200 Chemi-luminescent Imaging System (Tanon Science Technology, China).

## Results

### Isolation of maternal -inherited chlorophyll-deficient mutant (*cdm*)

**Fig. 1:**
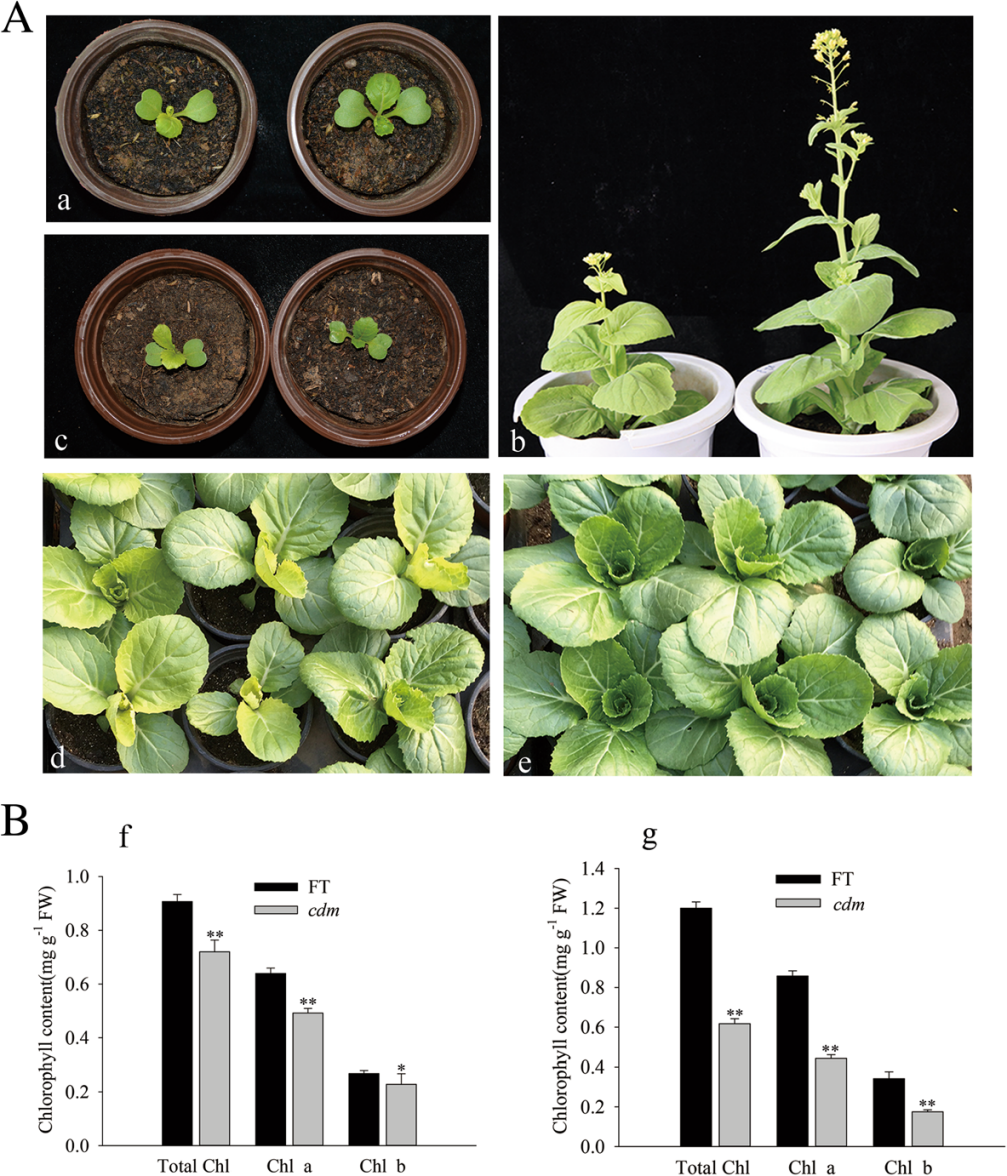
Identification of chlorophyll-deficient mutant (*cdm*) plants and their phenotypes at the seedling and mature stage. (**A**a, d, e) (left) and wild-type (‘FT’) plants at the seedling stage; (**A**b) *cdm* (left) and ‘FT’ plants at the mature stage; (**A**c) F1 plants derived from a cross of *cdm*× genetically distant inbred line (WZ) (left) and WZ × *cdm* (**B**) Chlorophyll content of *cdm* and ‘FT’ plants grown for 7 d and 25 d under long-day conditions. Error bars indicate one standard deviation of the mean (*n* = 5). Asterisks (**) indicate statistically significant differences between *cdm* and ‘FT’ at *P* < 0.01. fw, fresh weight

Microspore-derived regenerated plants (M0) were obtained from EMS mutagenesis on isolated microspores, and the variant plants were screened. All the plants were selfed to obtain M1 seeds. In M1 generation, all the new variant plants were also screened. A chlorophyll-deficient mutant (*cdm*) (Fig. 1a) was identified in the M0 generation and stably inherited in the progeny. Chlorophyll deficiency and reduced growth in *cdm* were more apparent in winter.

For genetic analysis, *cdm* plants were reciprocally crossed with ‘FT’ plants. The 376 F_1_ individuals were different, but consistent with the characteristics of female parent (Table 1), suggesting that *cdm* was a maternal mutant. For further verification, *cdm* plants were reciprocally crossed with a genetically-distant inbred Chinese cabbage line ‘WZ’ and found that only the maternal parent could transmit the mutant phenotype (Fig. 1a; Table 1).

**Table 1:** Genetic analysis of offspring derived from reciprocal crosses between chlorophyll-deficient mutant (*cdm*) and wild-type (‘FT’) or genetically-distant inbred line (WZ)

Cross	Number	Leaf color	
		Green	Yellow
*cdm* (selfed)	213	0	213
‘FT’ × *cdm*	174	174	0
*cdm* × ‘FT’	202	0	202
WZ × *cdm*	91	91	0
*cdm*× WZ	87	0	87

### Phenotypic characterization of *cdm*

**Fig. 2:**
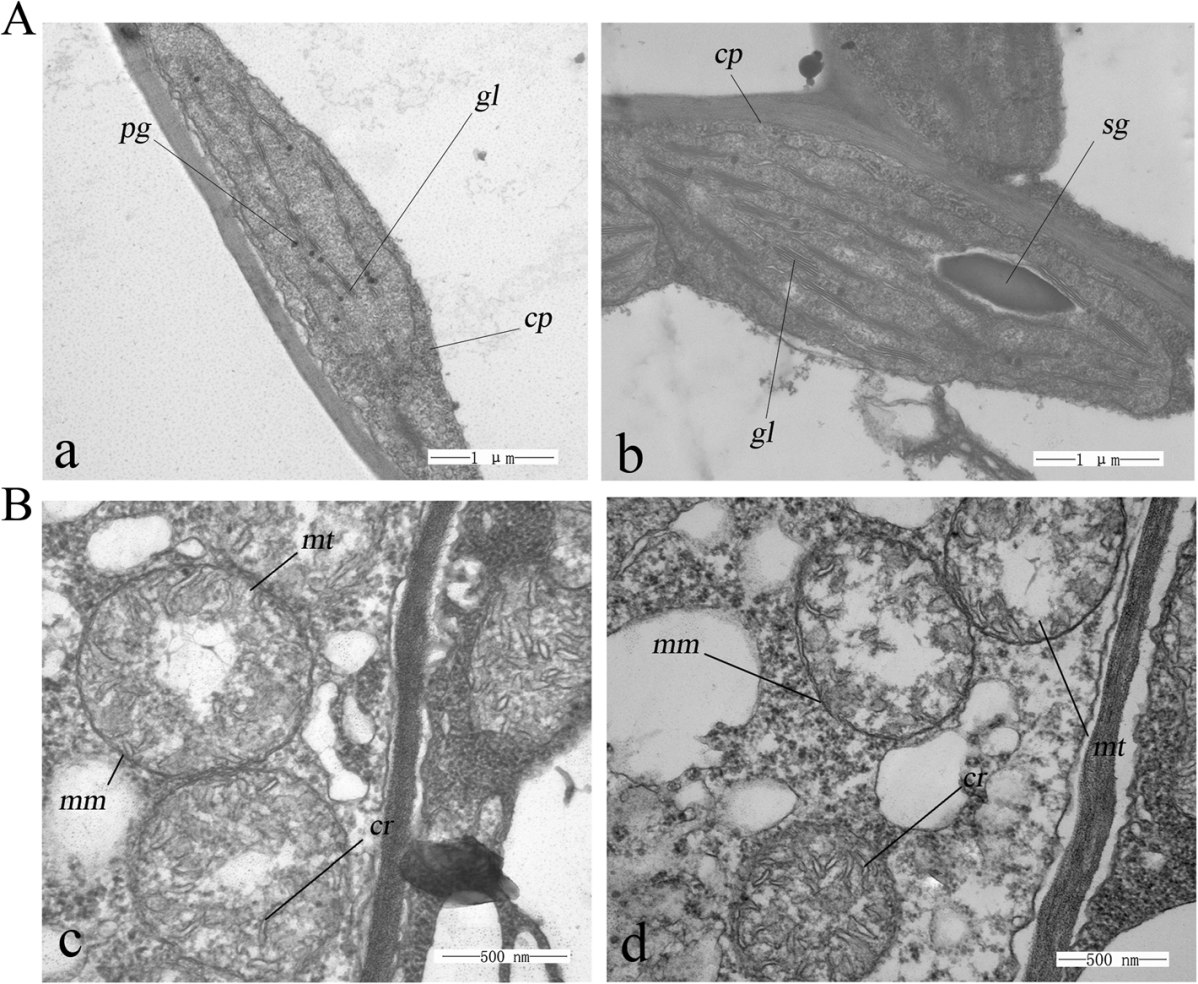
Chloroplast and mitochondrial ultrastructure of chlorophyll-deficient mutant (*cdm*) and wild-type (‘FT’) plants at the seedling stage (Chloroplast, × 30,000; Mitochondrion, × 60,000). (**A**a and b) Chloroplasts of *cdm* and ‘FT’. (**B**f and g) Mitochondria of *cdm*. cp, chloroplast; sg, starch grain; pg, plastoglobule; gl, grana lamella; mt, mitochondrion; mm, mitochondrial membrane; cr, mitochondrial crista. Scale bar, 1 μm. Bars = 1 μm (**A**) and 500 nm (**B**)

All Plants of *cdm* showed pale-yellow inner leaves and reduced growth. Consistently, the contents of total chlorophyll, chlorophyll a, and chlorophyll b were significantly lower in *cdm* at the seedling and mature stages compared with those in ‘FT’ (Fig. 1b). The results indicated that chloroplast development was severely impaired (Fig. 2a); however, no differences were observed in the mitochondria of *cdm* and ‘FT’ (Fig. 2b).

**Fig. 3:**
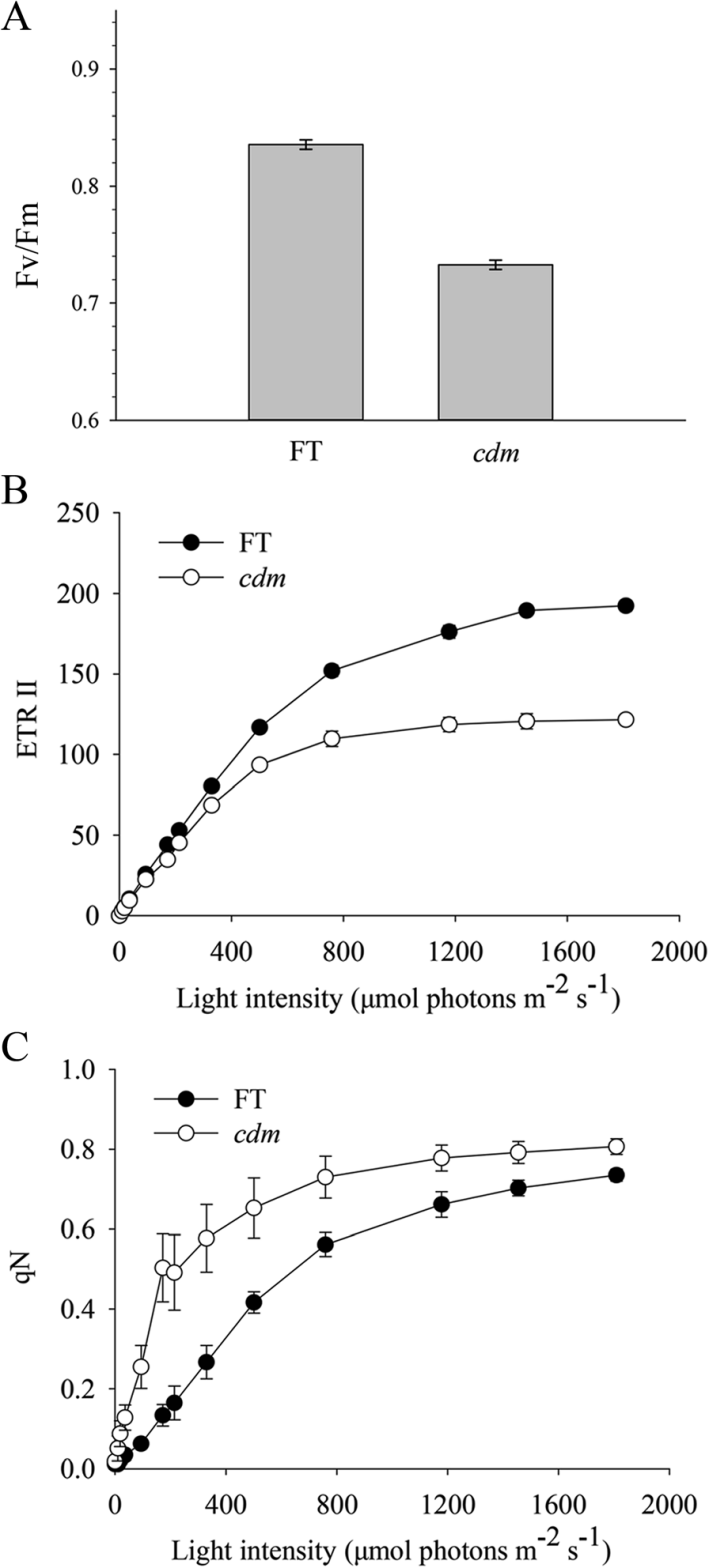
Photosynthetic activity in the leaves of chlorophyll-deficient mutant (*cdm*) and wild-type (‘FT’) plants. **a** Maximum quantum efficiency of PSII (Fv/Fm). **b** Light saturation curve of linear electron flux as calculated from the PSII yield (ETR II). **c** Non-photochemical quenching (NQP). Error bars indicate the SD (*n* = 4)

The PSII capacity of *cdm* was analyzed for various chlorophyll fluorescence parameters compared with that of ‘FT’. The results showed that Fv/Fm (Fig. 3a) and the electron transport rate (Fig. 3b) were lower in *cdm* than in ‘FT’, whereas qN (Fig. 3c) was higher in *cdm* than in ‘FT’, revealing PSII photo-inhibition and reduced photosynthetic efficiency in *cdm*.

### A missense mutation of chloroplast RPS4 in *cdm*

**Fig. 4:**
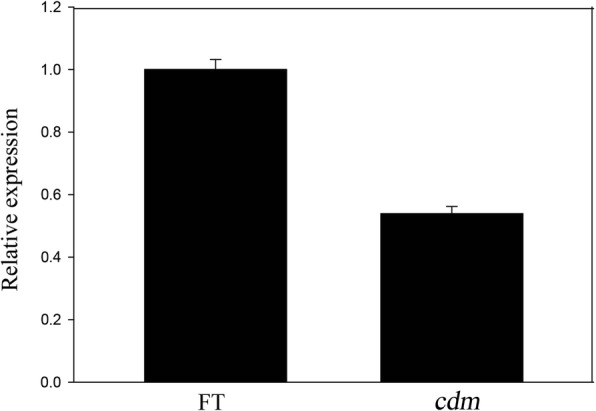
Expression of rps4 in chlorophyll-deficient mutant (*cdm*) and wild-type (‘FT’) plants detected by quantitative real-time PCR. Data are presented as means ± standard deviation of three biological replicates with three independent biological replicates

The chloroplast genome of *cdm* and ‘FT’ was re-sequenced and compared. Based on the inter-comparison of sequencing data and the reference genome, the frequency of A, T, C, and G on each site was recorded for the corresponding ratio in the chloroplast genome according to the sequence depth. All sites in the chloroplast genomes of *cdm* and ‘FT’ are presented in Additional file 2: Tables S2 and Additional file 3: Table S3. A site with a mutation ratio higher than 99% was detected in *cdm* after comparison of sequencing data (Table 2) and identified at nucleotide 44,398 of *rps4*, based on the reference genome and gene annotation as described by Wu et al. [64]. The A-to-C base substitution on this site was detected as a missense mutation that led to valine (Val) substitution for glycine (Gly) at residue 193, which is a highly conserved residue of RPS4 in plants. qRT-PCR was performed to detect any effects of the mutation on the expression of *rps4* and showed that the transcript level of RPS4 in *cdm* was lower than that in ‘FT’ (Fig. 4).

**Table 2:** Mutation sites in chlorophyll-deficient mutant (*cdm*) and wild-type (WT) compared with the reference genome

Location in Ref genome	Region	Mutation type	Reference	FT	*cdm*
265	trnH-psbA spacer	SNP	A	T	T
266	trnH-psbA spacer	SNP	A	T	T
267	trnH-psbA spacer	SNP	A	T	T
268	trnH-psbA spacer	SNP	A	T	T
20,164	rpoC1 coding region	SNP	C	T	T
44,398	rps4 coding region	SNP	A	A	C
66,083	psaJ-rpl33 spacer	SNP	A	T	T
66,084	psaJ-rpl33 spacer	SNP	G	T	T
66,085	psaJ-rpl33 spacer	SNP	A	C	C
66,086	psaJ-rpl33 spacer	SNP	A	T	T
116,457	psaC coding region	SNP	G	A	A

### Verifying the mutation in *rps4*

The inner leaves of *cdm* were uniformly pale green without any dark green sections, suggesting that the mutation might be homoplasmic and non-segregating. This assumption was supported by the A-to-C point mutation ratio in *rps4* that was higher than 99% and also by the sequence analysis of cpDNA from *cdm* and ‘FT’ which showed that the point mutation of *rps4* was present in different *cdm*. Therefore, the point mutation of *rps4* was homoplasmic in *cdm*.

### RPS4 structure

**Fig. 5:**
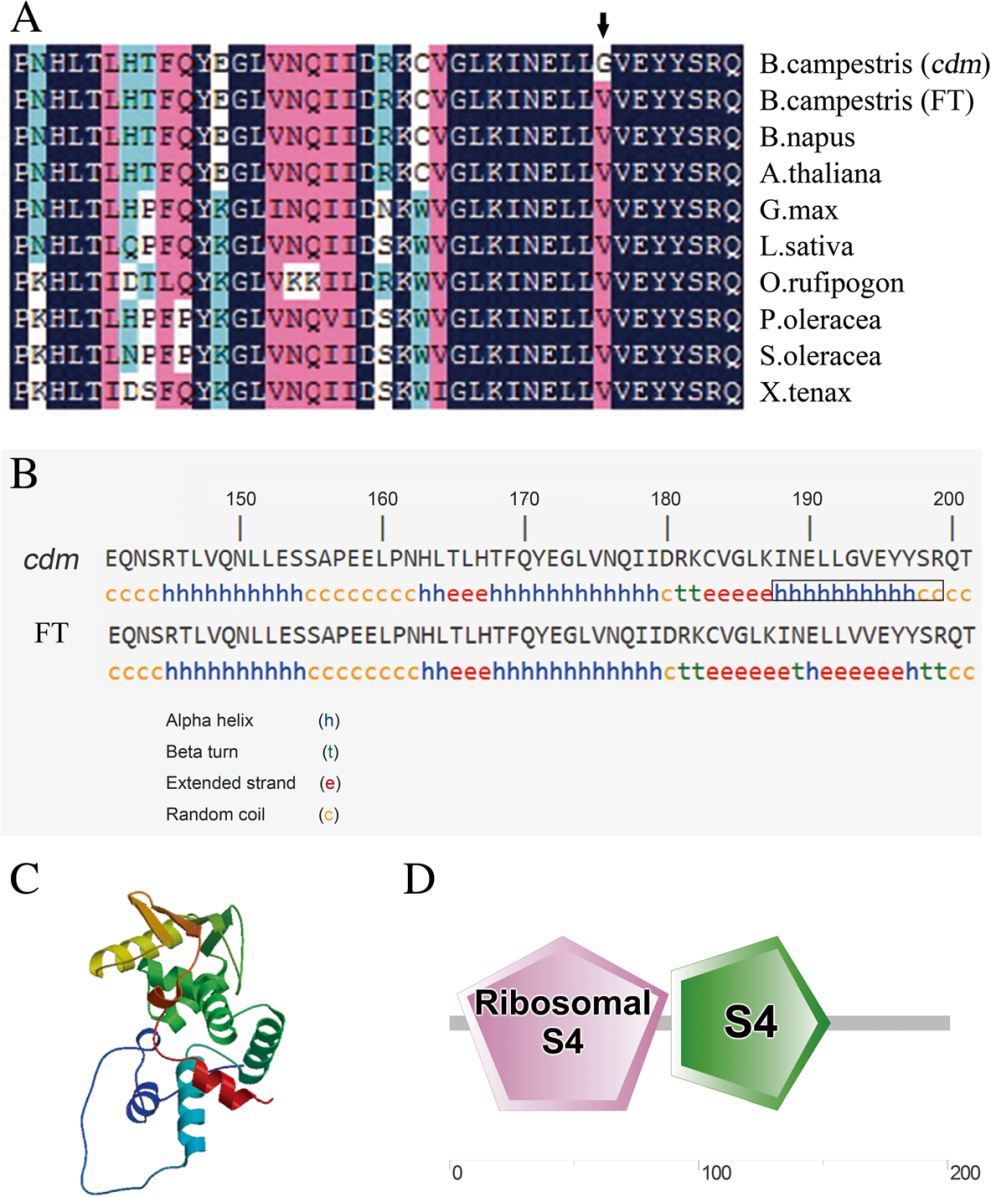
Partial sequence alignment and secondary structure prediction. **a** N-terminal domain sequence alignment of RPS4 in chlorophyll-deficient mutant (*cdm*) and wild-type (‘FT) of *Brassica campestris ssp., Arabidopsis thaliana*, *Brassica napus*, *Glycine max*, *Lactuca sativa*, *Oryza rufipogon*, *Portulaca oleracea*, *Spinacia oleracea*, and *Cyanophora paradoxa* (P23402). Vertical arrow indicates the substituted amino acid site. **b** Predicted secondary structure of the RPS4 N-terminal domain. Black box indicates sites altered secondary structure. **c** Predicted three-dimensional structure of RPS4. **d** Predicted protein domain of RPS4

RPS4 is a plastid ribosomal protein and a component of the plastid ribosome 30S small subunit. Its N-terminal region is highly conserved in higher plants (Fig. 5a). To study whether the amino acid substitution of RPS4 affected its secondary structure, the three-dimensional structure and the protein domain were predicted online at https://npsa-prabi.ibcp.fr/cgi-bin/npsa_automat.pl?page=/NPSA/npsa_sopma.html; https://swissmodel.expasy.org/; http://smart.embl-heidelberg.de/. The predicted secondary structure of RPS4 showed that the site of amino acid substitution was located at the extended strand in ‘FT’, whereas at the alpha helix in *cdm* (Fig. 5b). Thus, the predicted secondary structure of RPS4 in *cdm* was different from that in ‘FT’. However, the three-dimensional structure of RPS4 was the same in *cdm* and ‘FT’ (Fig. 5c). Zhang et al. [52] considered that the mutant site might not be analyzed by the prediction. RPS4 has two domains: the ribosomal protein S4 domain (3–88) and the S4 domain (89–153) (Fig. 5d). The ribosomal protein S4 domain binds to the ribosomal RNA and is composed of four helices that are discontinuous in sequence. The S4 domain probably mediates the binding to RNA. The amino acid substitution of RPS4 is not located in the two domains.

### Efficiency of chloroplast rRNA processing in *cdm*

**Fig. 6:**
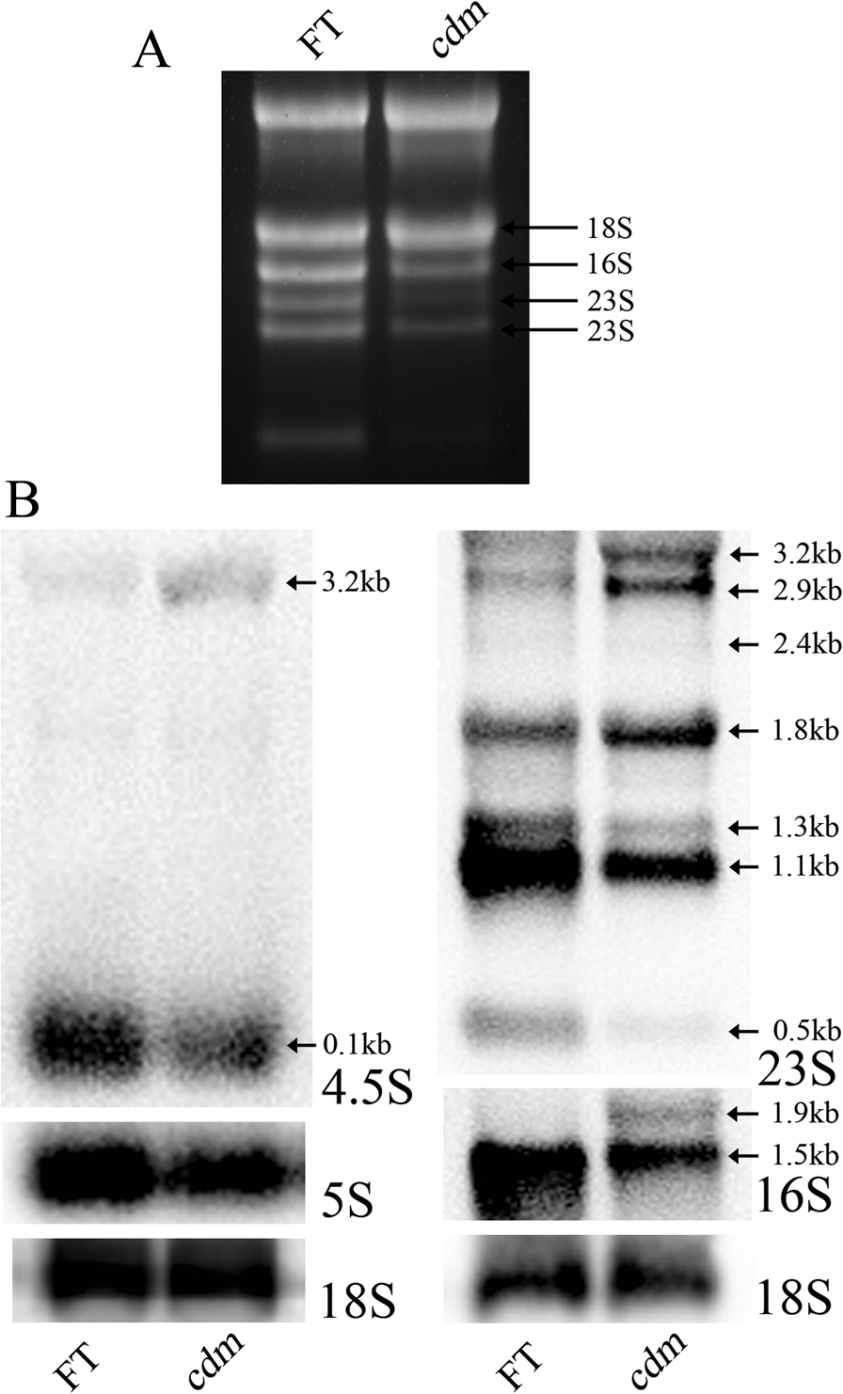
Chloroplast rRNA accumulation and processing in chlorophyll-deficient mutant (*cdm*) and wild-type (‘FT’) plants. **a** rRNA accumulation pattern in ethidium bromide-stained gel. **b** Northern blot analysis of chloroplast 23S, 16S, 5S, and 4.5S rRNAs and cytoplasmic 18S rRNA. Equal loading controls are shown in Additional file 5: Figure S2

To research whether the chloroplast rRNA processing was affected in *cdm* and *cdm* × ‘FT’, we investigated the pattern of total RNA by using denatured agarose gels electrophoresis. The results showed that the plastid rRNAs were significantly reduced in *cdm* and *cdm* × ‘FT’, compared with those in ‘FT’ and ‘FT’ × *cdm* (Fig. 6a, Additional file 4: Figure S1).

The plastid 70S ribosome is composed of a 50S subunit and a 30S subunit. The former is comprised by 23S rRNA, 5S rRNA, and 4.5S rRNA, 33 chloroplast ribosomal proteins, whereas the latter by 16S rRNA and 24 chloroplast ribosomal proteins. Chloroplast *rrn* operon is transcribed to generate large rRNA precursors, which form mature rRNAs after processing. For a more detailed analysis of the rRNA accumulation pattern of *cdm*, northern blot experiments were performed using gene-specific probes (Additional file 1: Table S1). The results showed that the abundance of mature 23S rRNA, 16S rRNA, 5S rRNA, and 4.5S rRNA was decreased in *cdm* compared with that in ‘FT’, whereas the precursors of 23S (3.2 kb, 2.9 kb, and 2.5 kb) rRNA, 16S (1.9 kb) rRNA, and 4.5S rRNA were over-accumulated in *cdm* (Fig. 6b). However, cytosolic 18S rRNA that used as a control showed similar levels in *cdm* and ‘FT’, indicating that cytosolic rRNA was not affected in *cdm* plants. Therefore, the mutation in *rps4* possibly impaired rRNA processing in *cdm*.

## Discussion

In the present study, we identified a chlorophyll-deficient mutant derived by EMS treatment on isolated microspores. The mutation showed non-Mendelian inheritance, indicating that it was located either in the chloroplast or in the mitochondrial genome. TEM studies showed that the ultrastructure of mitochondria was the same in *cdm* and ‘FT’, whereas that of plastids was abnormal in *cdm*. The plastome includes a conserved set of genes most of which are directly involved in photosynthesis or plastid translation [47, 49]. Thus, the mutation was likely in the plastome. The plastomes genomes of *cdm* and ‘FT’ were re-mapped to the reference genome of Chinese cabbage, revealing an A-to-C base substitution at nucleotide 44,398 of *rps4* with a mutation ratio higher than 99% that led to Val substitution for Gly in RPS4, which altered its secondary structure.

In our study, *cdm* showed uniformly pale green leaves and a slow growth rate. Almost all the chlorophyll-deficient cytoplasmic mutants are variegated [33], containing aberrant and normal plastids in distinct tissues of an individual plant, indicating heteroplasmy. However, all the inner leaves of *cdm* had a homogeneous pale-green color and extremely rare plant uniformity, suggesting homoplasmy and non-segregation. NGS showed that the mutation ratio of A-to-C base substitution was higher than 99% in *rps4*, verifying that the mutation was homoplasmic across all plastids. The pale green leaves of *cdm* plants were uniform, suggesting that the uniform mutant phenotype was due to a homoplasmic mutation. These results indicated that *rps4* was homoplasmic mutation, and might be responsible for the uniform chlorophyll deficiency of *cdm* plants.

The mechanism of EMS mutagenesis is based on the alkylation of guanine (G) residues, which primarily induces C-to-T changes and ultimately leads to an amino acid change or deletion [68]. The A-to-C base substitution in the present study did not correspond to a known mutagenesis mechanism, and thus, the homoplasmic mutation in *rps4* was assumed to be induced by a plastome mutator allele generated by EMS mutagenesis on isolated microspores. Plastome mutator alleles have been widely studied in *A. thaliana* [69], barley [27, 70], and the genus *Oenothera* [40, 71]. The cytoplasmic line 3 (CL3) in barley that selected from a barley plastid mutator genotype displays a homogeneous light green phenotype [27]. The plastome mutator can induce various plastome mutations in a homozygous recessive condition [33]. Since EMS treatment on isolated microspores can rapidly generate homozygous mutants, we speculated that a homozygous recessive nuclear mutation was generated, which was a plastome mutator that induced the point mutation in *rps4*.

Ribosomal proteins are essential components of the plastid ribosome and play different roles in rRNA processing and abundance. Mutations in plastid ribosomal proteins result in a range of developmental phenotypes, of which, some impair rRNA processing. Previous studies demonstrated that a mutation in *rps5*, *prps17*, and *prpl24* impairs rRNA processing in *A. thaliana* [51, 72]. Plastome-encoded ribosomal proteins, as well as their function, are highly conserved. For instance, the *rpl33* mutants in *E. coli* showed low-temperature tolerance [73, 74], whereas the *rpl33* mutants in tobacco showed decreased viability and growth under chilling stress [30]. RPS4 is required early in the assembly process, since it directly binds to the 16S rRNA in *Escherichia coli* [75] and is essential in tobacco [30]; thus, these studies support our assumption that mutation in *rps4* might affect the chloroplast ribosomal assembly process and impair plastid rRNA processing in *cdm*. By running denaturing agarose gels, we found that abundance of plastid rRNAs in *cdm* and *cdm* × ‘FT’ was decreased significantly than those in ‘FT’ and ‘FT’ × *cdm*. These results indicated that the impaired rRNA processing was maternal inherited. Northern blot data revealed a reduced abundance of mature 23S rRNA, 16S rRNA, and 4.5S rRNA, but over-accumulation of 23S rRNA, 16S rRNA, and 4.5S rRNA precursors in *cdm*. The missense mutation in RPS4 was the cause of aberrant rRNA processing in *cdm* that might be due to the reduced transcript level of RPS4 or the Val to Gly mutation in RPS4.

rRNA is stable only when integrated into ribosomal subunits; thus, rRNA abundance can serve as an indicator of the chloroplast ribosome content [76]. The ribosome is primarily composed of rRNA; thus, the aberrant processing of rRNA can affect ribosome function and consequently, plastid translation [47, 48, 77], which is indispensable in plant growth, development, and photosynthesis. Overall, our data suggested that the missense mutation in RPS4 might be the cause of aberrant rRNA processing, which affected plastid translation and resulted in chlorophyll deficiency and reduced plant growth.

## Conclusions

We first reported the identification of a homoplasmic plastome mutant in Chinese cabbage this study. The A-to-C point in the plastome-encoded *rps4* was associated with Chlorophyll deficiency and slow growth mutation in Chinese cabbage that impaired the rRNA processing and affected the ribosomal function and plastid translation.

## Additional files

Additional file 1: **Table S1.** Primers used in this study. (XLSX 11 kb)

Additional file 2: **Table S2.** Mutation sites in the plastome of wild type ‘FT’ compared with the reference genome. (FREQ 10682 kb)

Additional file 3: **Table S3.** Mutation sites in the plastome of *cdm* compared with the reference genome. (XLSX 9748 kb)

Additional file 4: **Figure S1.** rRNA accumulation pattern of *cdm*, ‘FT’, ‘FT’ × *cdm* and *cdm* × ‘FT’ in ethidium bromide-stainde gel. (TIF 21348 kb)

Additional file 5: **Figure S2.** Loading controls for RNA gel blotting. (TIF 16387 kb)

## Abbreviations

9AA: 9-aminoacridine hydrochloride
BrdU: 5-bromo-2′-deoxyuridine
EMS: Ethyl methanesulfonate
Fv/Fm: optional maximal photochemical efficiency of PS II
MNNG: methyl-nitro-nitrosoguanidine
NGS: Next-generation sequencing
NMU: N-nitroso-N-methyl-urea
RPS4: chloroplast ribosomal protein S4
SSC: saline sodium citrate
ФPSII: effective quantum yield of PSII, the photochemical quenching coefficient (QP), and qN non-photochemical Chl fluorescence quenching
