# Supplementary figures and images for: A missense mutation of plastid RPS4 is associated with chlorophyll deficiency in Chinese cabbage (Brassica campestris ssp. pekinensis)
Source: BMC Plant Biol. 2018 Jun 25;18:130. doi: 10.1186/s12870-018-1353-y (PMC6019835; doi:10.1186/s12870-018-1353-y)

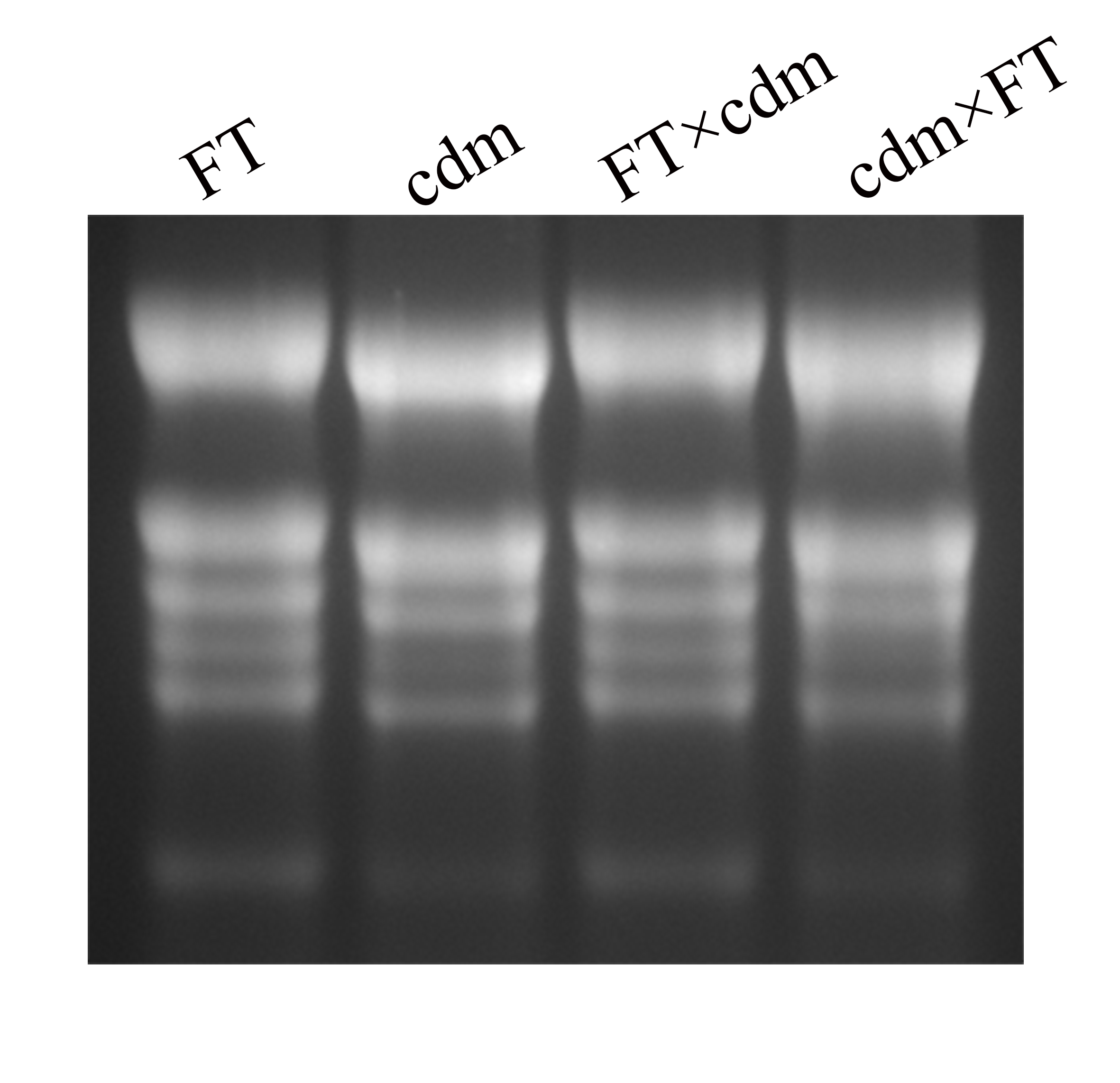

Supplement: Supplementary file 4 — Figure S1. rRNA accumulation pattern of cdm, ‘FT’, ‘FT’ × cdm and cdm × ‘FT’ in ethidium bromide-stainde gel. (TIF 21348 kb) [file 12870_2018_1353_MOESM4_ESM.tif]

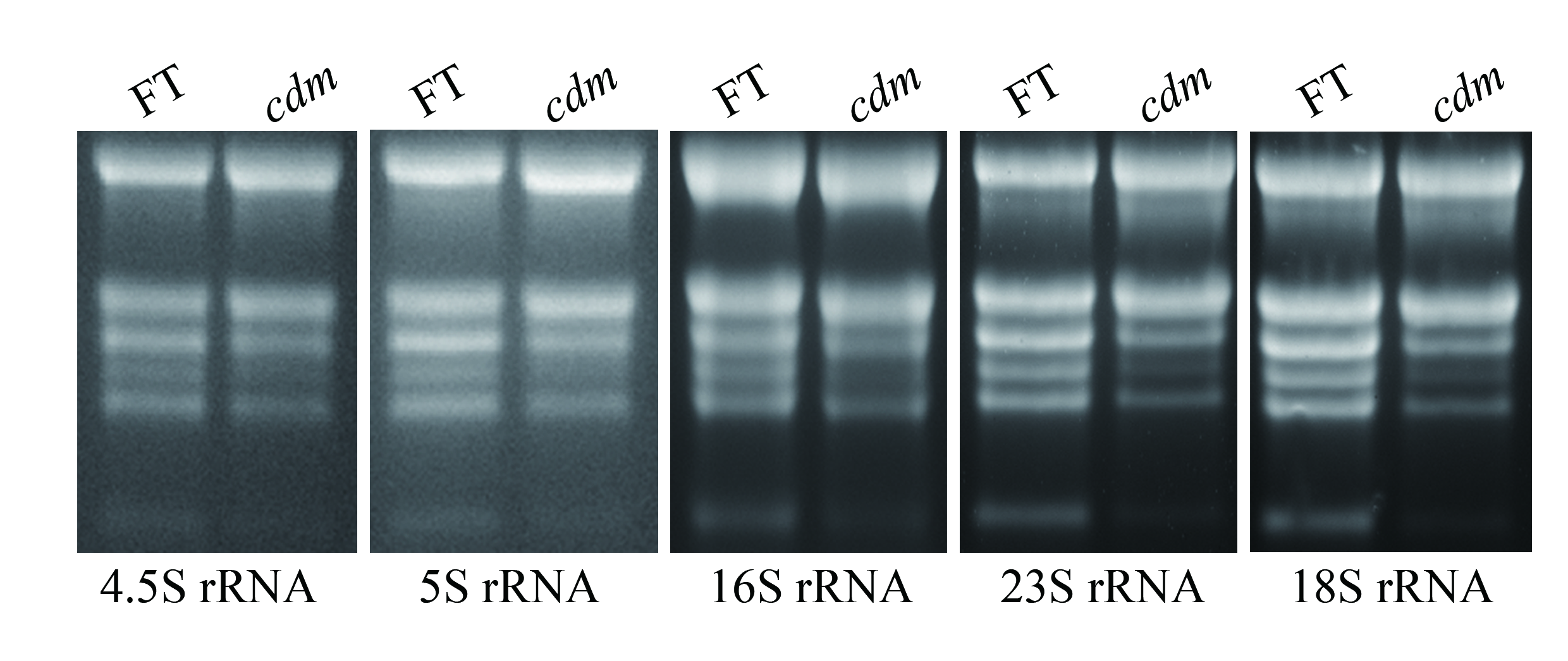

Supplement: Supplementary file 5 — Figure S2. Loading controls for RNA gel blotting. (TIF 16387 kb) [file 12870_2018_1353_MOESM5_ESM.tif]
